# The production of biodiesel from plum waste oil using nano-structured catalyst loaded into supports

**DOI:** 10.1038/s41598-021-03633-w

**Published:** 2021-12-16

**Authors:** Aasma Saeed, Muhammad Asif Hanif, Haq Nawaz, Rashad Waseem Khan Qadri

**Affiliations:** 1 Nano and Biomaterials Lab (NBL), Department of Chemistry, University of Agriculture, Faisalabad, 38040 Pakistan; 2 Institute of Horticultural Sciences, University of Agriculture, Faisalabad, 38040 Pakistan

**Keywords:** Chemistry, Energy science and technology

## Abstract

The present study was undertaken with aims to produced catalyst loaded on low-cost clay supports and to utilize plum waste seed oil for the production of biodiesel. For this purpose, Bentonite–potassium ferricyanide, White pocha-potassium ferricyanide, Granite-potassium ferricyanide, Sindh clay-potassium ferricyanide, and Kolten-potassium ferricyanide composites were prepared. Transesterification of plum oil under the different conditions of reactions like catalysts concentrations (0.15, 0.3 and 0.6 g), temperature (50, 60, 70 and 80 °C), reaction time (2, 4 and 6 h) and oil to methanol ratio (1:10) was conducted. The maximum biodiesel yield was recorded for Bentonite–potassium ferricyanide composite. This composite was subjected to calcination process to produce Calcinized bentonite–potassium ferricyanide composite and a further improvement in biodiesel amount was recorded. The fuel quality parameters of all biodiesel samples were in standard range. Gas chromatographic mass spectrometric analysis confirmed the presence of oleic and linoleic acids in the plum seed oil. The characterization of composite was done using FTIR, SEM and EDX. Two infrared bands are observed in the spectrum from 1650 to 1630 cm^−1^ indicates that the composite materials contained highly hydrogen bonded water. The presence of surface hydroxyls groups can also be confirmed from FTIR data. SEM image clearly show the presence of nano-rods on the surface of Granite-potassium ferricyanide and Kolten-potassium ferricyanide composites. Another interesting observation that can be recorded from SEM images is the changes in surface characteristic of Bentonite–potassium ferricyanide composite after calcination (at 750 °C, 1 atm for 4 h). Calcinized bentonite–potassium ferricyanide composite found to contain more nano rod like structures at its surface as compared to Bentonite–potassium ferricyanide composite which contained spherical particles. EDX data of Bentonite–potassium ferricyanide composite and Calcinized bentonite–potassium ferricyanide composite show that after calcination carbon and oxygen was reduced. The other lost volatile compounds after calcination were of Na, Mg, Al, Si, and S. The XRD spectrum of pure bentonite showed the average crystal size of 24.46 nm and calcinized bentonite of 25.59 nm. The average crystal size of bentonite and potassium ferricyanide composite and its calcinized form was around 33.76 nm and 41.05 nm, respectively.

## Introduction

Clay based minerals materials are commonly found around us and have applications as catalysts in the organic synthesis since long time. Several types of clay-based catalysts have designed and applied for use in organic synthesis including ion-exchanged, acidic, and basic clay catalysts. The use of abundant and commonly available natural kaolin is one such example that has been used for preparing precursors and catalytic supports. The clay mineral’s original crystalline structure can be altered by means of different treatments in a controlled way to enhance their use as catalyst. The treatments that are most commonly performed for modification are done with pillaring, impregnation, intercalation, and acids that are effective in modifying the surface area, in optimization of the active sites, and to facilitate the attachment of the reagent molecules to active sites through the mesopores (average diameter of 20 to 500 Å)^1^. The inorganic salts in the form of heterogenous catalysts such as KF impregnated on γ-Al_2_O_3_, Zn–Al(O) Ca–Al mixed oxides, CaO, and CaO–Fe_3_O_4_ supports has been successfully utilized in the production of biodiesel. Bentonite is an absorbent swelling clay consisting mostly of montmorillonite that been used previously to enhance catalytic activity of the catalysts to produce biodiesel^2–8^.

Plum seeds are waste product that is generated from juice, food processing and cosmetic industries. Plum seeds produce over 50% of oil, this fruit is an important feed stock to produce biodiesel oil. The quality of biodiesel depends on the composition profile of fatty acids produced from the feedstock and is also important when we decide future biodiesel production possibilities^9,10^. The plum processing industry can be an interesting source of alternative, inexpensive oily raw materials for biodiesel synthesis due to the world production and processing of plums and high oil content in grain. Total global plum production in 2013 amounted to 11.5 million tons and about 12 million tons in 2020. The oil content in plum grains (PK) ranges from 32% to even 45.9%, which is similar to sunflower and rapeseed oil (about 40%) but is higher than in soybean (about 20%). Biodiesel is best alternative available today to conventional diesel. Everyday introduction of improved methods of production in new alternative feedstocks are ever rapidly cementing biodiesel strong position as a mainstream alternative. There are several benefits of biodiesel including local production, rural development, environment friendly fuel and economically sound product as compared to conventional diesel. Why biodiesel did not attain that popularity which was expected due to its well-known so many benefits. Biodiesel production is viable only if produced from non-food crops. To make it further popular and competitive it is necessary to use non-food crops. It will not only reduce production costs but will also end food verses fuel debate^11–16^. There are several clays including Bentonite, White pocha, Kolten and Sindh clays which are used in ceramic industry and available at very low cost. Granite stone powder is a waste product of stone processing industries and is a material available free of cost. All these materials were selected in the present study to prepare composite supports to hold catalyst potassium ferricyanide to produce biodiesel from non-edible waste seed oil of plum. The various parameters affecting the yield were tested for optimum production of biodiesel. The percentage biodiesel yield depends on different parameters like effect of catalysts concentrations, effect of reaction time and temperature, and oil to methanol ratio were optimized during the present study. The quality of biodiesel samples was monitored through standard procedures including iodine value, cetane number, specific gravity, density, and acid value. Finally, advanced instrumental techniques were used for characterization of catalysts, supports and produced biodiesel of zeolite with different catalysts and composite supports were prepared and also characterized.

## Materials and methods

### Materials and oil extraction

Plum stones were collected from local market, juice, and fruit shops of Faisalabad City, Pakistan. Plum stones were broken to obtain seeds. The collected seeds were dried to remove moisture^17^. Oil was extracted from crushed dried seeds by using of expeller machine. The extracted oil was allowed to stand for 24 h to settle down of impurities and particles. Free fatty acid contents (FFA) of oil was determined using acid–base titration. Bentonite, White pocha, Kolten and Sindh clays were obtained from Ceramic Industry, Gujranwala, Pakistan. All chemical used in the present study were of analytical grade.

### Preparation of catalyst and transesterification of oil

Bentonite potassium ferricyanide composite was prepared by mixing 25 g of potassium ferricyanide and 25 g of bentonite clay in 250 mL distilled water. The mixture was stirred for 5 min at 100 rpm and filtered. The obtained solid material was dried at 60 °C in an electric oven. By following similar procedure potassium ferricyanide catalyst was mixed with different support materials including White pocha, Granite, Sindh clay, and Kolten. Bentonite–potassium ferricyanide composite have shown maximum biodiesel yield during the present study. To improve catalytic activity of Bentonite–potassium ferricyanide composite further, this composite was subjected to at 750 °C for 4 h.

Transesterification of plum oil under the different conditions of reactions like catalysts concentrations (0.15, 0.3 and 0.6 g), temperature (50, 60, 70 and 80 °C), reaction time (2, 4 and 6 h) and oil to methanol ratio (1:10) was conducted. Magnetic stirring was maintained at 300 rpm during all experiments. Glycerol was formed as a byproduct during biodiesel production. The upper biodiesel was separated from glycerol and washed with hot water until clear biodiesel layer was obtained. The biodiesel quality was accessed by the determination of density, specific gravity, pH, saponification value, acid value, cetane number, iodine value, free fatty acids contents and acid value^13,18–20^.

### Characterization of plum oil

GC–MS (gas chromatographic-mass spectrometric analysis) was conducted for the quantification of methyl esters present in the biodiesel. For this purpose, three samples were selected. GC–MS analysis was performed on a Perkin Elmer Clarus 600 GC System, fitted with an Elite-5MS capillary column (30 m × 0.25 mm i.d. × 0.25 μm film thickness; maximum temperature, 350 °C), coupled to a Perkin Elmer Clarus 600C MS. Ultra-high purity helium (99.999%) was used as a carrier gas at a constant flow of 0.2 ml/min. The injection, transfer line and ion source temperatures were 220, 200 and 200 °C, respectively. At the ionizing energy of 70 eVthe data was collected from 10 to 600 m/z by using 0.1 μL of sample with 50:1 spilt ratio. The temperature program for oven was as follows: 35 °C holds for 10 min, 10 °C/min 200 °C hold for 10 min. The unknown compounds were identified by the use of NIST 2011 (v.2.3 and Wiley, 9th edition).

## Results and discussion

### Optimization of biodiesel yield

**Figure 1:**
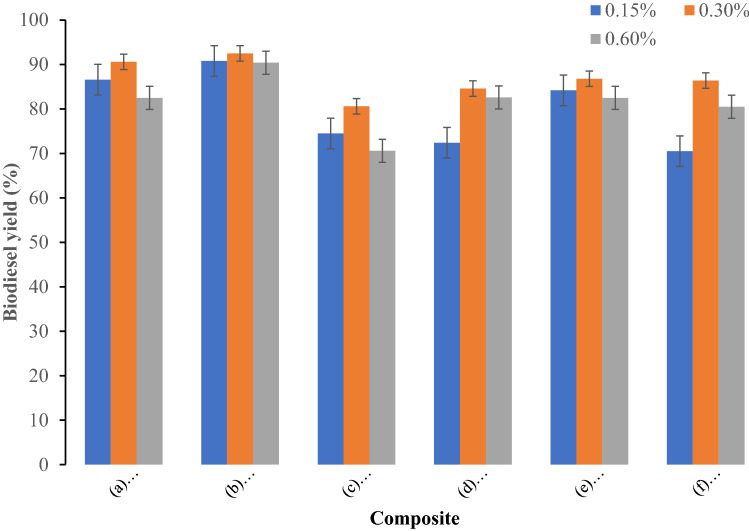
Effect of catalyst concentration on yield of biodiesel produced from plum waste oil (**a**) Bentonite–potassium ferricyanide composite, (**b**) Calcinized bentonite–potassium ferricyanide composite, (**c**) White pocha-potassium ferricyanide composite, (**d**) Granite-potassium ferricyanide composite, (**e**) Sindh clay-potassium ferricyanide composite, (**f**) Kolten-potassium ferricyanide composite.

**Figure 2:**
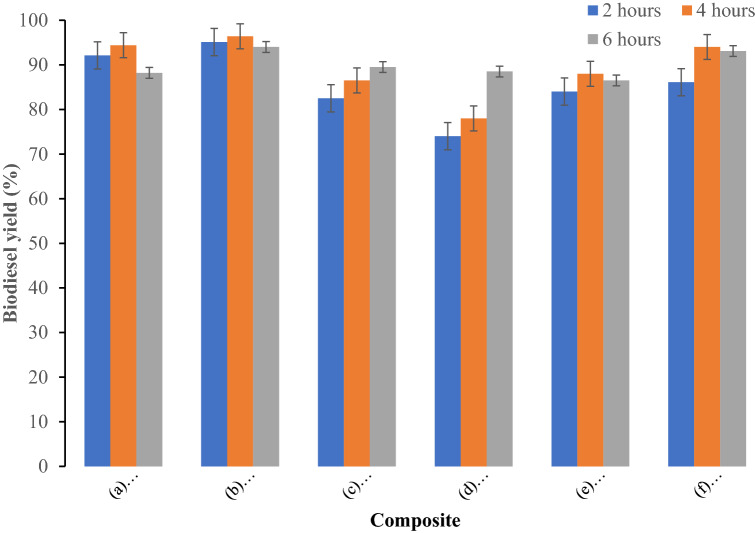
Effect of reaction time on yield of biodiesel produced from plum waste oil (**a**) Bentonite–potassium ferricyanide composite, (**b**) Calcinized bentonite–potassium ferricyanide composite, (**c**) White pocha-potassium ferricyanide composite, (**d**) Granite-potassium ferricyanide composite, (**e**) Sindh clay-potassium ferricyanide composite, (**f**) Kolten-potassium ferricyanide composite.

**Figure 3:**
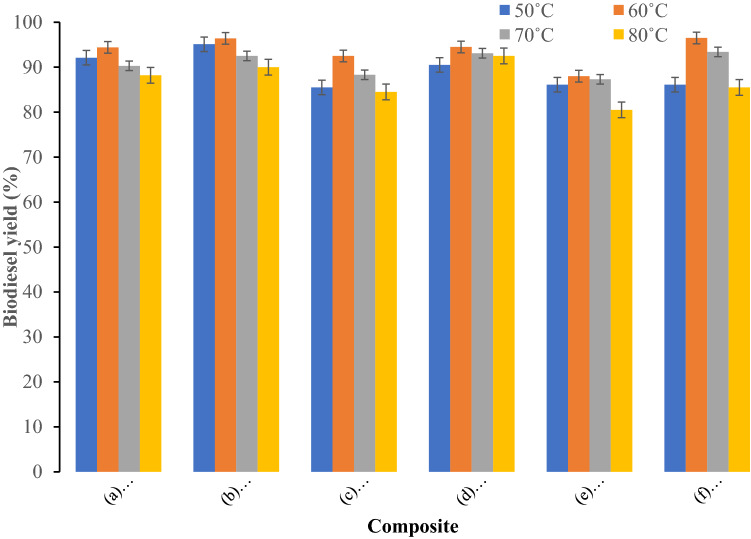
Effect of temperature on yield of biodiesel produced from plum waste oil (**a**) Bentonite–potassium ferricyanide composite, (**b**) Calcinized bentonite–potassium ferricyanide composite, (**c**) White pocha-potassium ferricyanide composite, (**d**) Granite-potassium ferricyanide composite, (**e**) Sindh clay-potassium ferricyanide composite, (**f**) Kolten-potassium ferricyanide composite.

The plum oil was transesterified into fatty acid methyl esters in the presence of following composite materials: (a) Bentonite–potassium ferricyanide composite (b) Calcinized bentonite–potassium ferricyanide composite (c) White pocha-potassium ferricyanide composite (d) Granite-potassium ferricyanide composite (e) Sindh clay-potassium ferricyanide composite (f) Kolten-potassium ferricyanide composite. Effect of catalyst concentration on yield of biodiesel produced from plum waste oil was studied at three catalyst concentrations 0.15, 0.3 and 0.6%. The biodiesel yield increased on increasing catalyst amount from 0.15 to 0.30%. The maximum biodiesel yield for all composite catalysts was obtained at 0.30%. A further increase in the catalyst amount decreased the biodiesel yield. The decrease in the biodiesel yield at increased catalyst concentrations might be due to increase in the viscosity of reaction mixture (Fig. 1). The effect of reaction times on biodiesel yield was evaluated from to 2 to 6 h (Fig. 2) at methanol to oil molar ratio 8:1, 0.3% catalyst, and 60 °C reaction temperature. The maximum biodiesel yield for (a) Bentonite–potassium ferricyanide composite (b) Calcinized bentonite–potassium ferricyanide composite (e) Sindh clay-potassium ferricyanide composite and (f) Kolten-potassium ferricyanide composite was obtained after 4 h. A further increase in the reaction time resulted in the loss of biodiesel yield may be due to breakdown of fatty acid methyl esters. However, (c) White pocha-potassium ferricyanide composite and (d) Granite-potassium ferricyanide composite have produced maximum of biodiesel after 6 h of reaction time. The determination of an optimum time to produce biodiesel is essential as it contributes to calculate the cost on pilot and commercial scales. The impact of transesterification reaction temperature was studied on three temperatures (50, 60, 70 and 80 °C) by keeping other variables constant as follows: reaction time of 4 h, catalyst amount 0.3%, and methanol to oil ratio of 10:1 (Fig. 3). The rate of transesterification reaction increased as the reaction temperature increased from 50 °C to 60 °C. On increasing reaction temperature from 60 to 80 °C, a decrease in the biodiesel yield was observed. Although, it is expected that biodiesel yield increases with the temperature, however, increasing temperature above 60 °C could result in the decrease of biodiesel yield due gasification of methanol^21^.

Among all used composites as catalysts, the maximum biodiesel yield was obtained for Bentonite–potassium ferricyanide. The calcination of Bentonite–potassium ferricyanide composite have further increased the biodiesel yield. The highest biodiesel yield observed using calcinized Bentonite–potassium ferricyanide composite was due to increase in the average crystal size (as supported by XRD results) that has provided comparatively greater surface area for reactants for successful conversion into products. The XRD spectrum of pure bentonite showed the average crystal size of 24.46 nm and calcinized bentonite of 25.59 nm. The average crystal size of bentonite and potassium ferricyanide composite and its calcinized form was around 33.76 nm and 41.05 nm, respectively.

### Determination of fuel properties

The estimated values of various fuel quality parameters are tabulated in Table 1. Biodiesel density is important parameters as the fuel working in the fuel injector system and engine is strongly related to density value^22^. Amount of weight comprised in a unit volume is referred as density. Denser the oil more the energy it contains. Standards used to measure density of biofuels are 3675/12185 in European Union and D1298 in USA and are measured at reference temperature of 15 or 20 °C. Density which is measured by comparing with water’s density is called relative density. The density of biodiesel measured relatively necessary for calculating the conversion of mass to volume and for determination of flow rate (Sanford et al., 2009). The densities of different biodiesel samples determined in the present study was between 0.856 to 0.877 kg/L. The recommended range of density lies between 0.86 and 0.90 g/cm^3^ by EN 14214:2003 for a B100 type biodiesel. All biodiesel samples have density in the recommended range. These results reveal that produced biodiesel may be suitable for optimal performance.

**Table 1:** Determination of biodiesel fuel properties.

Catalyst	Density (g/cm^3^)		
	0.15%	0.3%	0.6%
(a) Bentonite–potassium ferricyanide composite	0.864	0.861	0.862
(b) Calcinized bentonite–potassium ferricyanide composite	0.877	0.866	0.860
(c) White pocha-potassium ferricyanide composite	0.867	0.871	0.875
(d) Granite-potassium ferricyanide composite	0.870	0.861	0.876
(e) Sindh clay-potassium ferricyanide composite	0.877	0.873	0.874
(f) Kolten-potassium ferricyanide composite	0.868	0.869	0.869

	Acid values (mg of KOH/g)		
(a) Bentonite–potassium ferricyanide composite	0.7846	0.1562	0.1121
(b) Calcinized bentonite–potassium ferricyanide composite	0.5454	0.1553	0.8413
(c) White pocha-potassium ferricyanide composite	0.5854	0.6854	0.1983
(d) Granite-potassium ferricyanide composite	0.8212	0.1883	0.1892
(e) Sindh clay-potassium ferricyanide composite	0.1134	0.5034	0.1894
(f) Kolten-potassium ferricyanide composite	0.1883	0.5423	0.5521

	Iodine values (g I_2_/100 g)		
(a) Bentonite–potassium ferricyanide composite	156.3	150.2	154.6
(b) Calcinized bentonite–potassium ferricyanide composite	145.2	140.2	143.2
(c) White pocha-potassium ferricyanide composite	154.7	152.3	152.1
(d) Granite-potassium ferricyanide composite	158.1	155.2	153.8
(e) Sindh clay-potassium ferricyanide composite	158.2	159.1	154.3
(f) Kolten-potassium ferricyanide composite	168.3	160.1	143.2

	Saponification value (mg KOH/g)		
(a) Bentonite–potassium ferricyanide composite	130.13	134.22	140.66
(b) Calcinized bentonite–potassium ferricyanide composite	122.11	110.33	120.51
(c) White pocha-potassium ferricyanide composite	186.23	100.98	185.13
(d) Granite-potassium ferricyanide composite	105.78	129.03	103.78
(e) Sindh clay-potassium ferricyanide composite	180.53	103.78	179.52
(f) Kolten-potassium ferricyanide composite	165.52	165.49	164.51

	Cetane number		
(a) Bentonite–potassium ferricyanide composite	67.80	78.53	67.89
(b) Calcinized bentonite–potassium ferricyanide composite	55.58	85.43	53.46
(c) White pocha-potassium ferricyanide composite	56.45	69.76	78.54
(d) Granite-potassium ferricyanide composite	56.45	87.58	92.47
(e) Sindh clay-potassium ferricyanide composite	56.23	63.45	57.34
(f) Kolten-potassium ferricyanide composite	55.67	78.54	77.42

Acid value is defined “as the number of milligrams of potassium hydroxide (KOH) required to neutralize the free fatty acid in oil”. The acid values of all biodiesel samples produced in the present study were in the standard range. Iodine value is “the measure of the total degree of unsaturation, and it provides useful guidance for preventing various problems in engines”. The iodine value tells about stability and the presence of double bonds in the fatty acid methyl esters^23–25^. The iodine values measured during the present study ranged from 140.2 to 168.3 g I_2_/100 g. Catalyst concentration is an important factor that affect the iodine value of produced biodiesel samples.

Saponification value is “the amount of alkali required to saponify a given quantity of oil sample, which is expressed as the number of milligrams of KOH required to saponify 1 g of oil sample and is inversely proportion to the molecular weight of fatty acid of the biodiesel”^22^. The saponification value of plum oil biodiesel ranged from 103.78 to 186.23 mg/g. Cetane number is a fuel quality parameter related to the ignition delay time and combustion quality. According to UNE-EN 14214 (2003) specification, biodiesel should have minimum Cetane number of 51, while ASTM D6751-02 assigns 47 as the minimum cetane number for biodiesel. The cetane number of all biodiesel sample were greater than 50. Cetane values obtained in the present study has higher value as compared to the previous study on soya bean oil that ranged from 45 to 60^22^.

### Gas chromatographic analysis (GC–MS analysis)

The fatty acid composition of plum seed oil is given in the Table 2. The chemical composition of biodiesel determines its fuel stability, while the stability of the fatty acid methyl ester depends on its number of double bonds, polyunsaturated fatty acids, which are more susceptible to oxidation than the fatty acid having single bond^26^. The major fatty acids present in the plum oil were oleic acid and linoleic acid. According to a previous study, the oils having fatty acids with more than 15 carbon atoms as major components could be explored to produce good quality biodiesel^13^.

**Table 2:** Fatty acid profile of plum waste oil.

Fatty acid	Percentage composition (%)
Linolenic acid	0.80
Stearic acid	0.50
Palmitic acid	3.20
Oleic acid	71.2
Linoleic acid	23.6

### Characterization of composite supports

**Figure 4:**
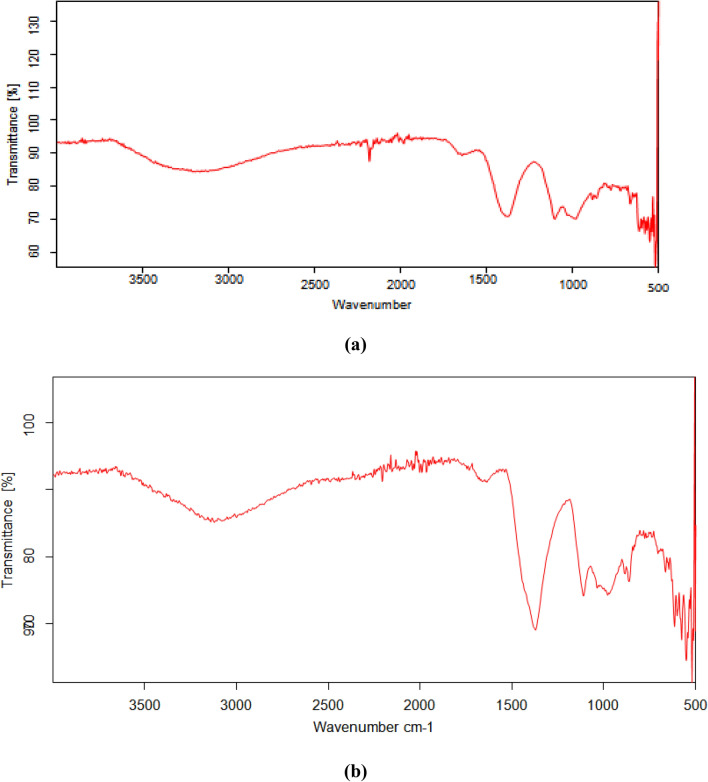

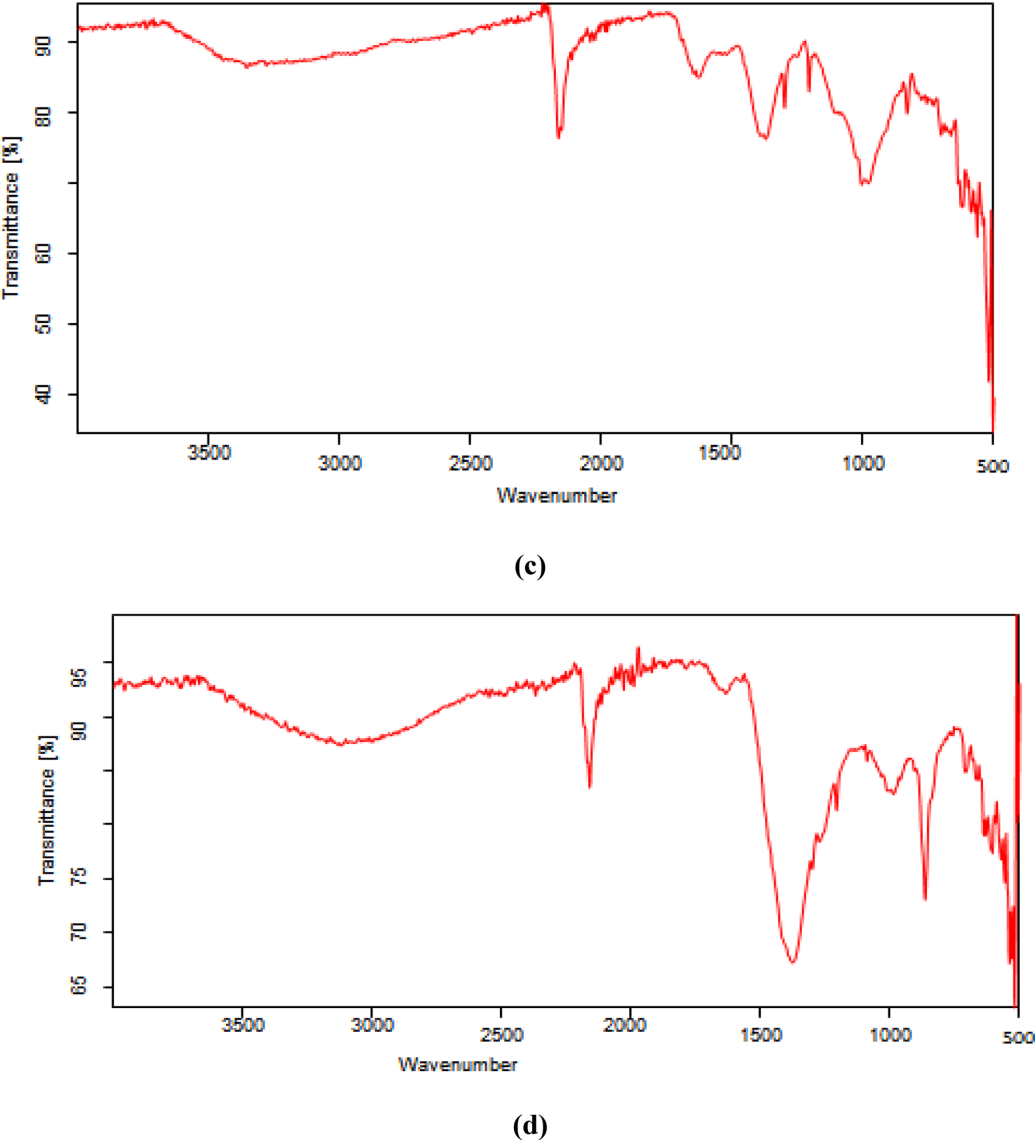

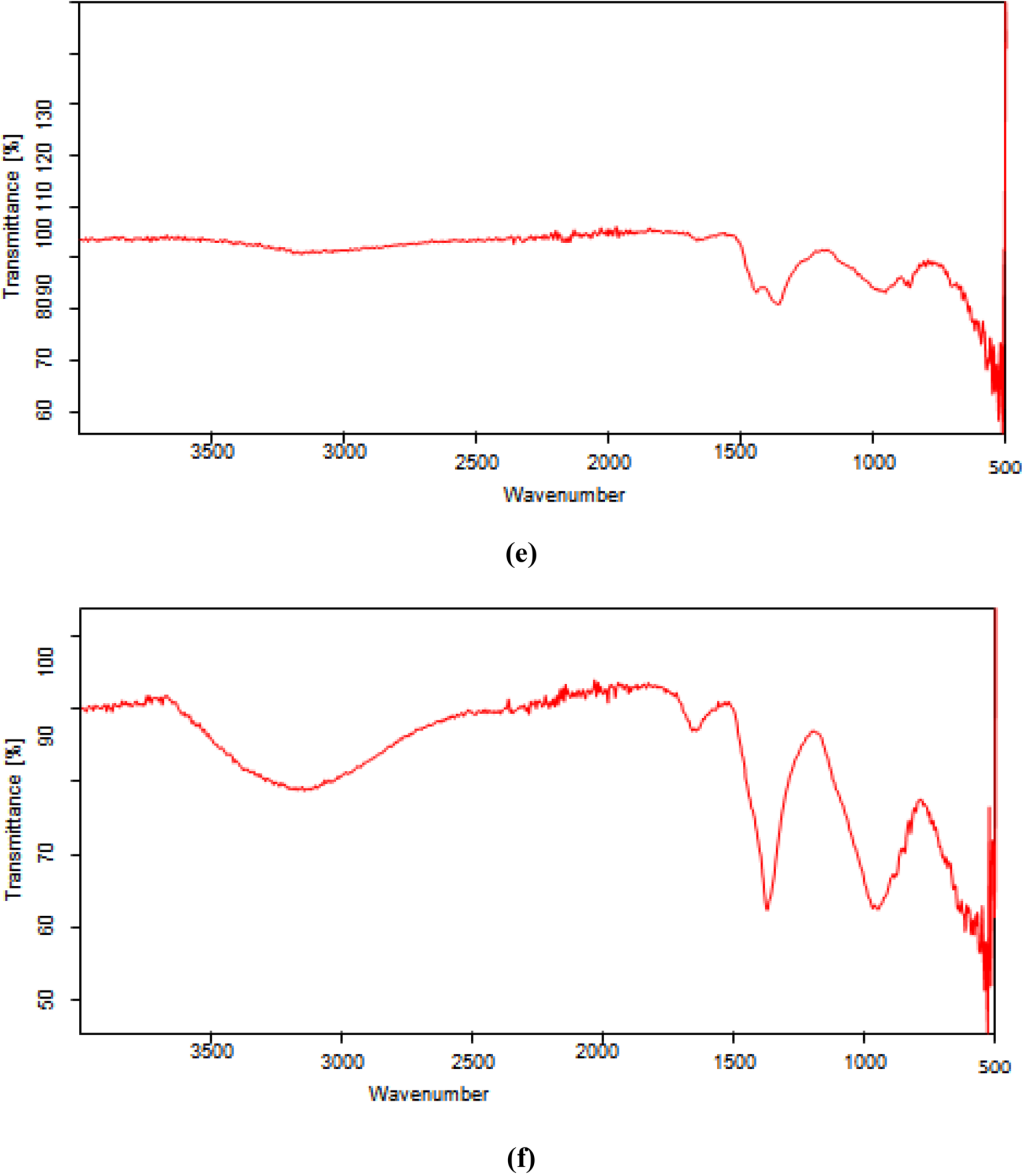
FTIR various composites (**a**) Bentonite–potassium ferricyanide composite, (**b**) Calcinized bentonite–potassium ferricyanide composite, (**c**) White pocha-potassium ferricyanide composite, (**d**) Granite-potassium ferricyanide composite, (**e**) Sindh clay-potassium ferricyanide composite, (**f**) Kolten-potassium ferricyanide composite.

FTIR spectra of various composites used in the present study including (a) Bentonite–potassium ferricyanide composite (b) Calcinized bentonite–potassium ferricyanide composite (c) White pocha-potassium ferricyanide composite (d) Granite-potassium ferricyanide composite (e) Sindh clay-potassium ferricyanide composite (f) Kolten-potassium ferricyanide composite are presented in the Fig. 1. The peaks in functional group region (4000–1500 cm^−1^) are characteristic of specific kinds of bonds, and therefore can be used to identify whether a specific functional group is present. The peaks in fingerprint region (1500–400 cm^−1^) arise from complex deformations of the molecule. They may be characteristic of molecular symmetry, or combination bands arising from multiple bonds deforming simultaneously. It can be seen from Fig. 1 that overall FTIR spectra of all composites significantly vary from each other although peaks in some regions matches too. However, frequencies of absorbing functional groups were different in all cases. A broad band of different intensities seen in the all-composite catalytic materials in the range of 3550–3200 cm^−1^ is due to O–H stretching vibrations. A clear C≡C band in the range of 2300–2100 cm^−1^ was observed for Bentonite–potassium ferricyanide composite, Calcinized bentonite–potassium ferricyanide composite, White pocha-potassium ferricyanide composite and Granite-potassium ferricyanide composite. Sindh clay-potassium ferricyanide composite and Kolten-potassium ferricyanide composite did not show any clear band. However, a weak band was present for C≡C. The bands in the region of 1650–1600 cm^−1^ (C=C stretching), 1650–1580 cm^−1^ (N–H bending), 1550–1500 cm^−1^ (N–O stretching) and 1000–1200 cm^−1^ (C–O stretching) were observed in all composite materials, however, they have shown variable transmittance intensities. A clear difference between bentonite–potassium ferricyanide composite and calcinized bentonite–potassium ferricyanide composite band intensity can be seen in FTIR spectra (Fig. 4). Calcination, which refers to the heating of inorganic materials to remove volatile components. The release of volatile matter during calcination minimizes internal shrinkage in later processing steps that can lead to the development of internal stresses and, eventually, cracking or warping. Calcination treatment is an integral part during fabrication and activation of the heterogeneous catalysts^27^.

The OH-bending shows vibrations of the inner surface OH groups were observed at 913 cm^−1^ and that of the surface OH groups near 936 cm^−1^; the surface hydroxyls are associated with additional bands near 701 and 755 cm^−1^. Iron-bearing composite materials show typical of bands due to Fe(AlFeOH) at 865–875 cm^−1^ and compressing at 3607 cm^−1^^28^. Two infrared bands are observed in the spectrum from 1650–1630 cm^−1^ indicates that the composite materials contained highly hydrogen bonded water^29^. The band just above 3600 cm^−1^ (at 3620 cm^−1^) corresponds to the "inner hydroxyls" located on the plane common to octahedral and tetahedral sheets. The vibration of the "outer hydroxyls" located on the surface and along the broken edges of composites may be attributed to bands recorded at 3668 and 3652 cm^−1^. Adsorption of ions and complexes on clay minerals is considered to occur as a result of surface complexation, ion exchange, electrostatic and hydrophobic interaction^30^. The adsorption capacity modes on mineral surfaces are primarily divided into complexes of the outer sphere and inner-sphere surface. In general, in the inner-sphere complexes, chemical interactions are stronger than in the outer-sphere complexes. The mobility of ionic species in the environment influences these differences in binding strengths^31–33^.

**Figure 5:**
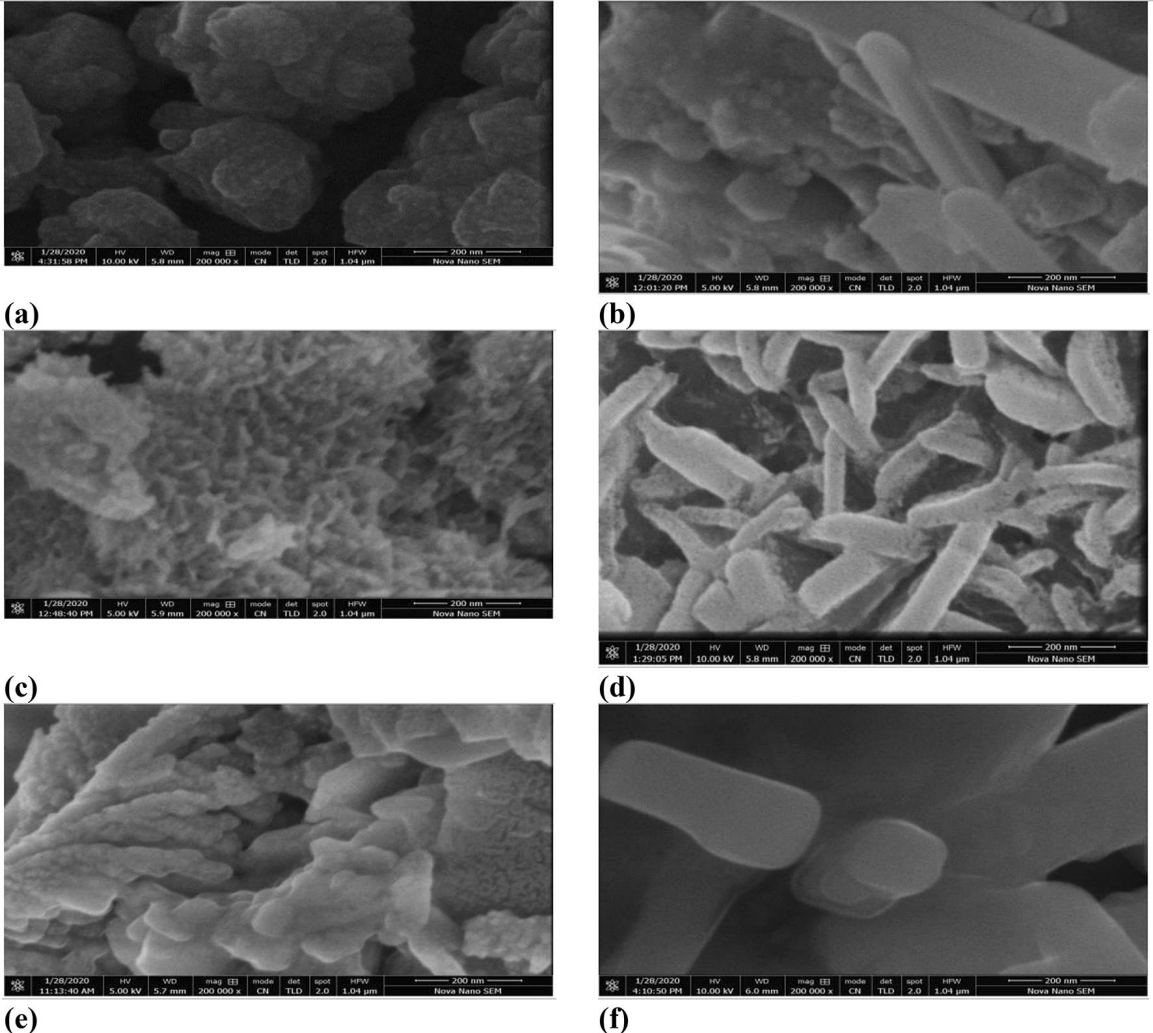
SEM images of various composites at 200 nm (**a**) Bentonite–potassium ferricyanide composite, (**b**) Calcinized bentonite–potassium ferricyanide composite, (**c**) White pocha-potassium ferricyanide composite, (**d**) Granite-potassium ferricyanide composite, (**e**) Sindh clay-potassium ferricyanide composite, (**f**) Kolten-potassium ferricyanide composite.

Scanning electron microscopy (SEM) images were recorded to study surface morphology of different catalytic materials (Fig. 5). SEM image clearly show the presence of nano-rods on the surface of Granite-potassium ferricyanide composite and Kolten-potassium ferricyanide composite. Another interesting observation that can be recorded from SEM images is the changes in surface characteristic of Bentonite–potassium ferricyanide composite after calcination. Calcinized bentonite–potassium ferricyanide composite found to contain more nano rod like structures at its surface as compared to Bentonite–potassium ferricyanide composite which contained spherical particles. In broader sense, SEM images show that catalyst loaded composite materials surface particles were different in size and of variable shape^34^.

**Figure 6:**
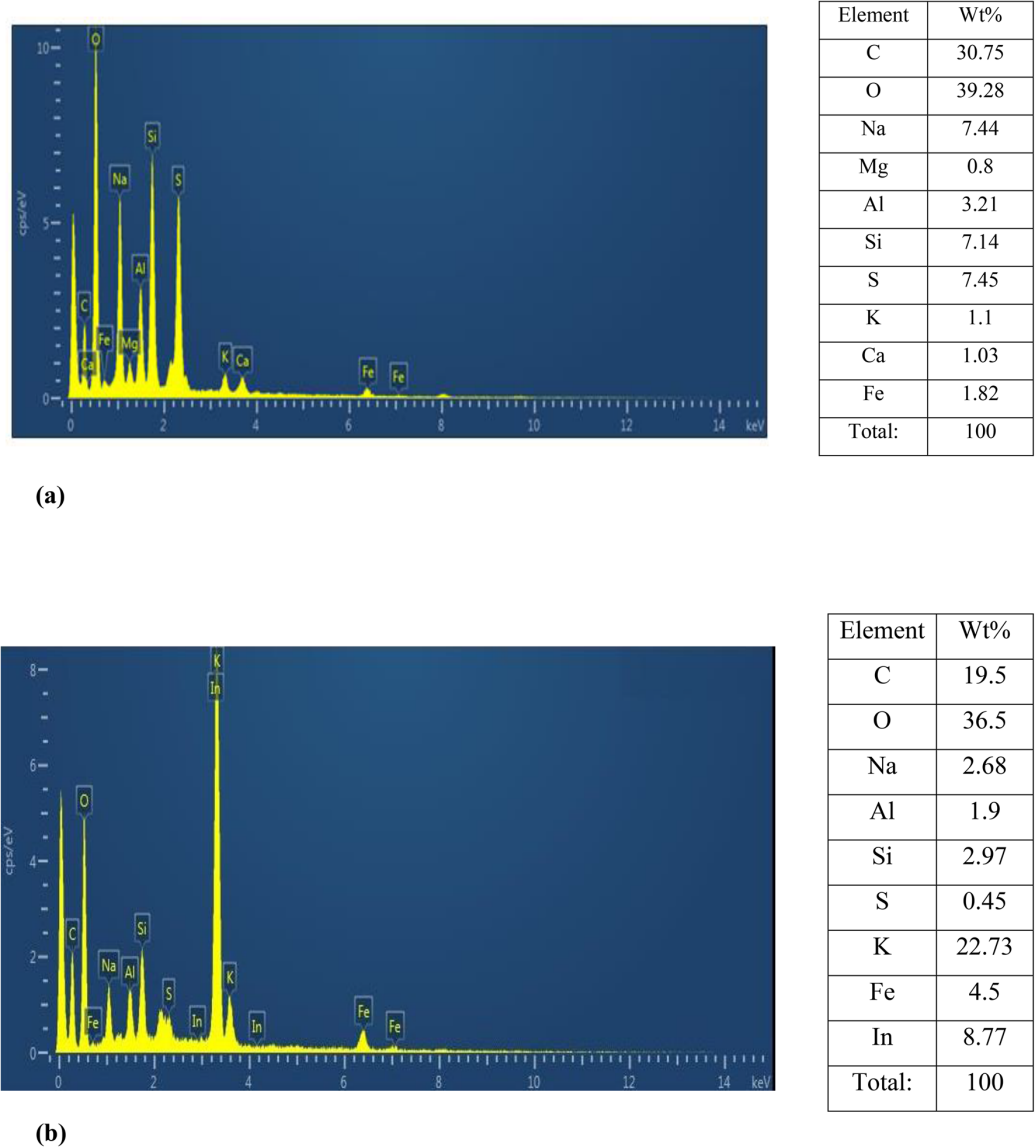

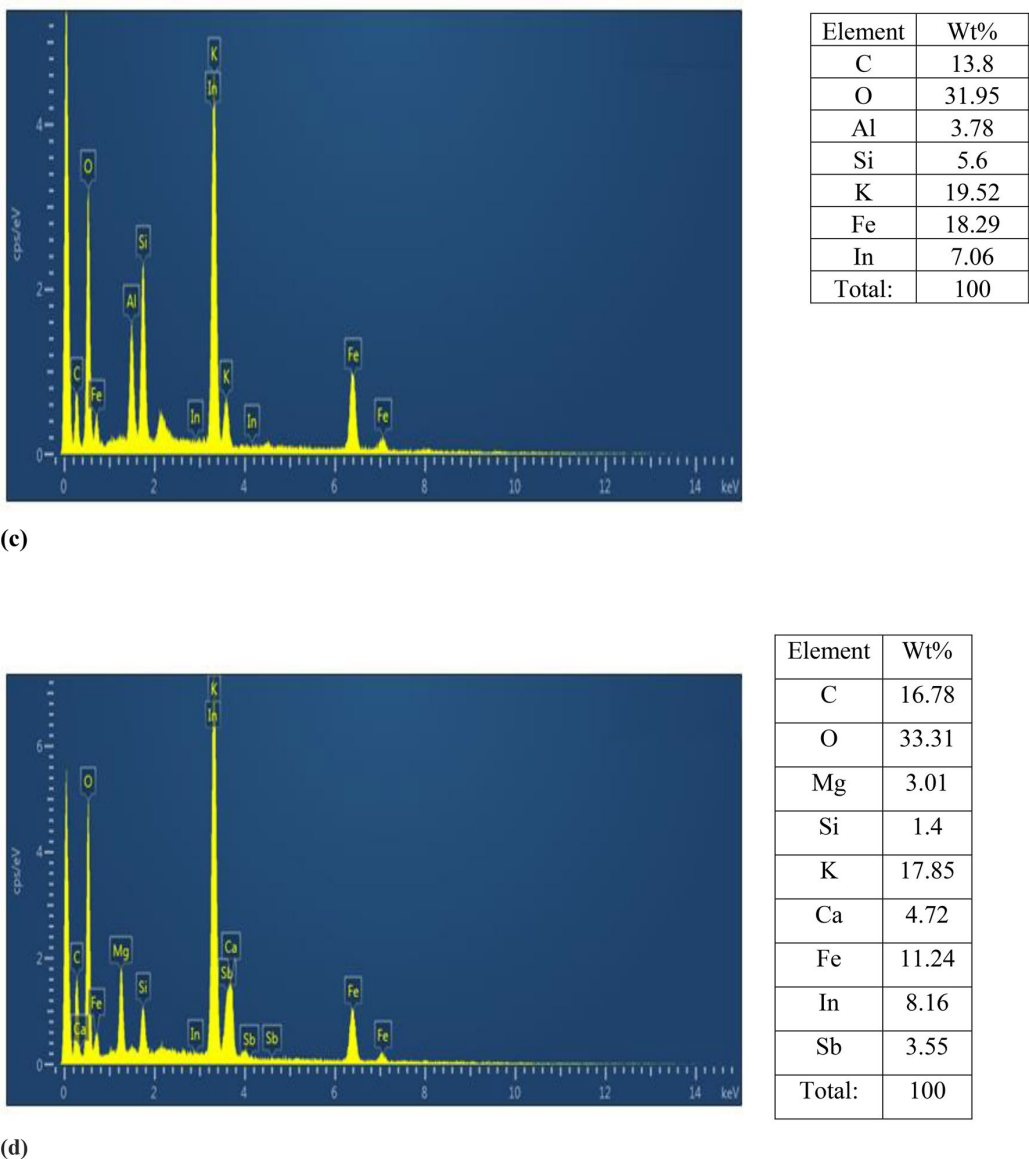

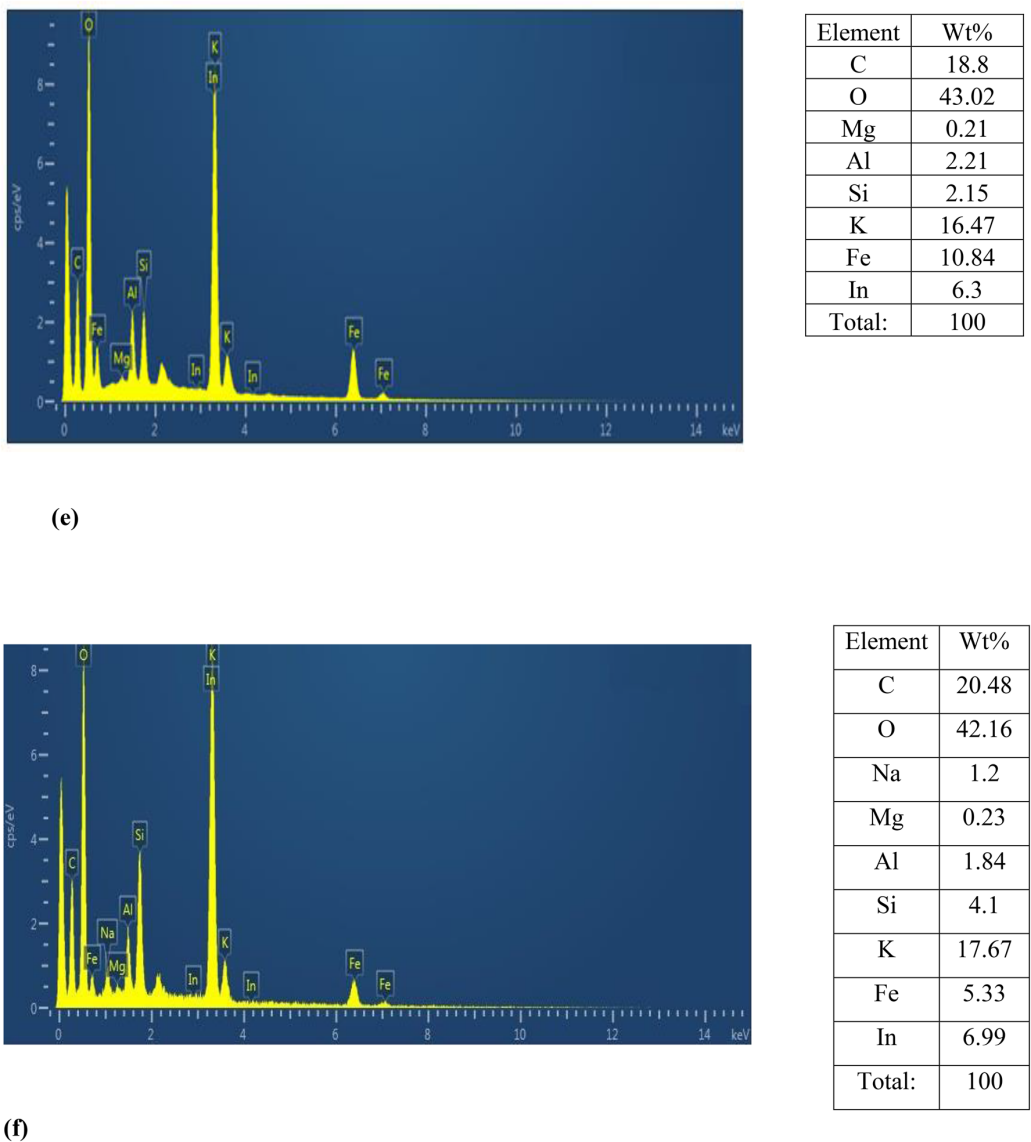
EDX images (**a**) Bentonite–potassium ferricyanide composite, (**b**) Calcinized bentonite–potassium ferricyanide composite, (**c**) White pocha-potassium ferricyanide composite, (**d**) Granite-potassium ferricyanide composite, (**e**) Sindh clay-potassium ferricyanide composite, (**f**) Kolten-potassium ferricyanide composite.

Energy-dispersive X-ray spectroscopy (EDX) is a powerful tool for the analysis of fine-grained clay mineral components, and Al-pillared clays in particular. It can be seen that Al, Si, Co, Ni and Fe are the primary elements of the untreated clay. The exchange process resulted in an increase in the composite's aluminum, iron and nickel content. EDX spectra for all composite materials were recorded and is presented in Fig. 6. The S, Al, S, Mg, P, Ca and O were recorded in the element analysis EDX, and various peaks of Fe and C of carbon with Fe_3_O_4_ elements were also observed. Oxygen was present as a major element and shows the presence of most other compounds as oxygen derivatives. Bentonite–potassium ferricyanide composite was calcinized to produce Calcinized bentonite–potassium ferricyanide composite. EDX data of Bentonite–potassium ferricyanide composite and Calcinized bentonite–potassium ferricyanide composite show that after calcination carbon and oxygen was reduced. The other lost volatile compounds after calcination were of Na, Mg, Al, Si, and S. Within the clay structure, the content of these elements was not constant, and the pillaring process impacted it. The increase of Al–Si is due to presence of Al_13_ poly cation in the Al-pillared clay particle. Which can be seen in the curves, the reflectance of the non-treated clay is lower than that of the particles containing Al-integrated clays and in the considered spectral region varies between 50 and 60%. The findings also showed that there are more EM waves reflected from the Al-exchanged clays than those from the Al-pillared ones. This could be due to the contribution of electrical dipoles or multiple reflection phenomena in Al-exchanged clays from the front face of the first layer^35^.

**Figure 7:**
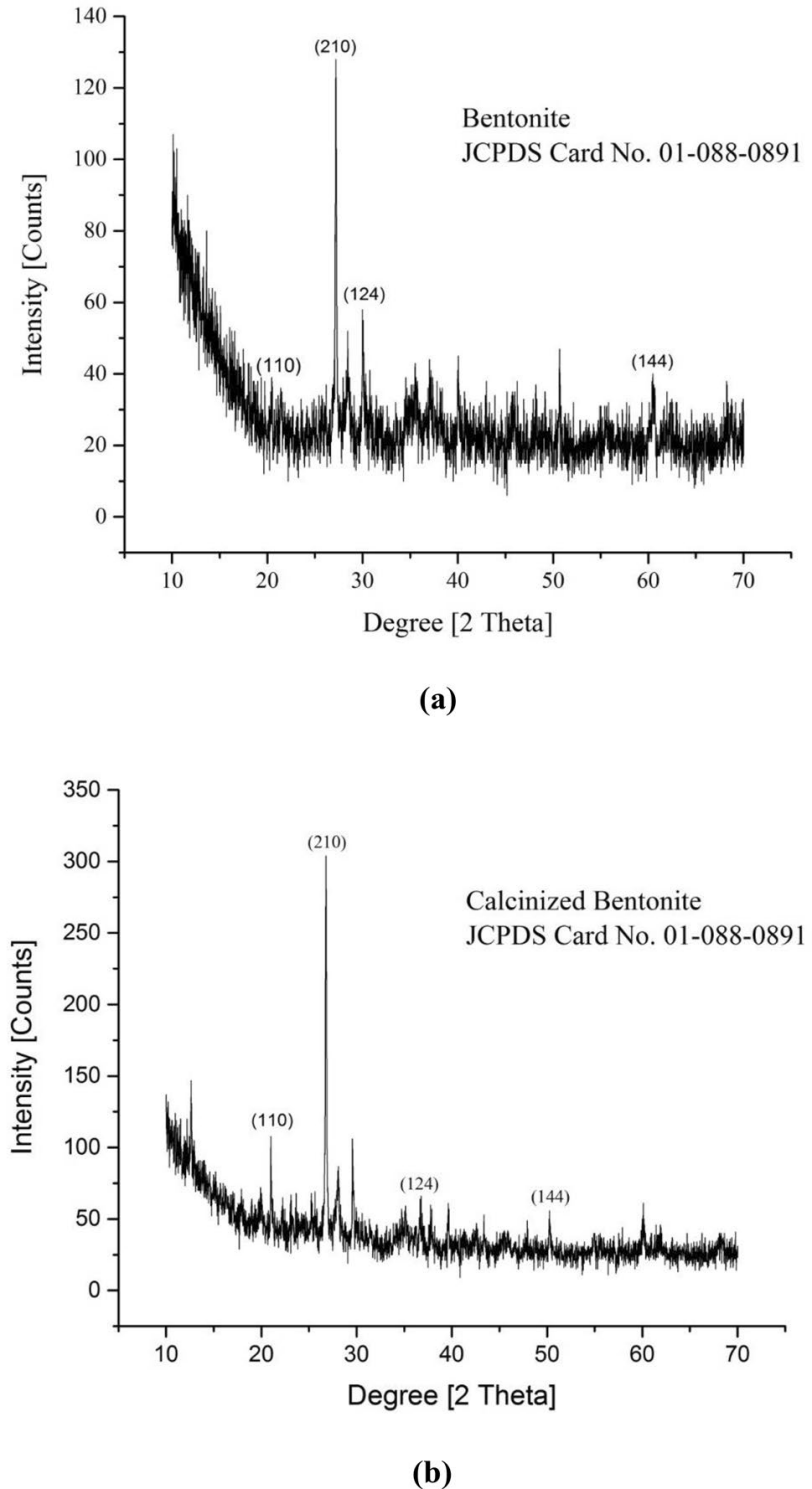

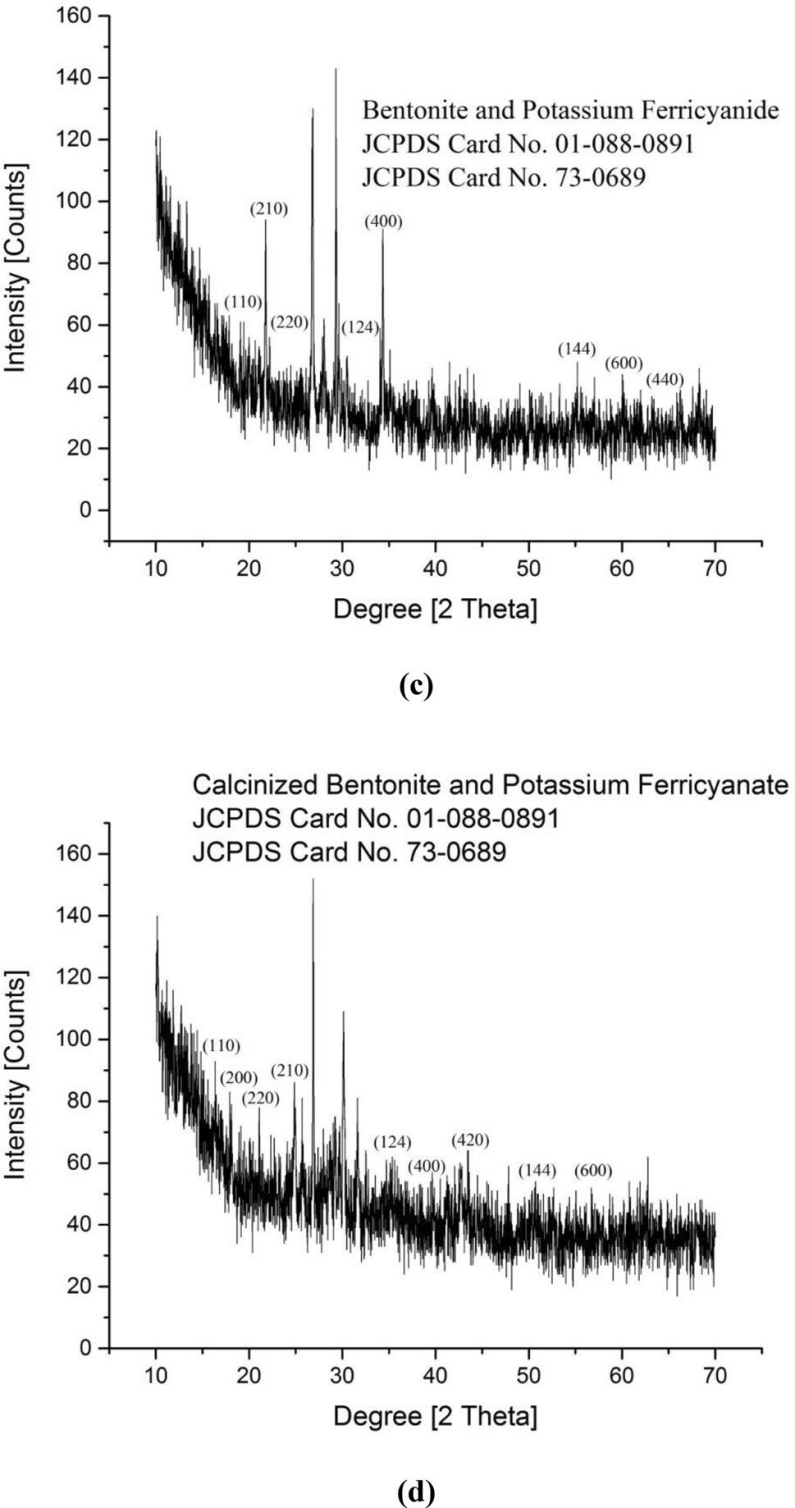
XRD spectrum of (**a**) bentonite, (**b**) calcinized bentonite, (**c**) bentonite and potassium ferricyanide composite, (**d**) calcinized bentonite and potassium ferricyanide composite.

The optimized catalytic materials that have shown highest transesterification ability were subjected to XRD studies (Fig. 7) including bentonite, calcinized bentonite, bentonite, and potassium ferricyanide composite and calcinized bentonite and potassium ferricyanide composite (Tables 3, 4, 5 and 6). Bentonite is a potential adsorbent and a swelling clay having montmorillonite as a major constituent. It generally consists of silicon dioxide (SiO_2_), aluminum trioxide (Al_2_O_3_), ferric oxide (Fe_2_O_3_), calcium oxide (CaO), sodium oxide (Na_2_O), magnesium oxide (MgO) and potassium oxide (K_2_O). The bentonite consists of silicon (Si), aluminum (Al), oxygen (O), sulphur (S) and carbon (C) as major chemical elements. The XRD spectrum of pure bentonite showed the sharp characteristic peaks at diffraction angle 2θ as follows: 21.42° (110), 27.20° (210), 30.01° (124), and 60.44° (144). These results are in close agreement with the international standard compound under JCPDS Card No. 01–088-0891. The average crystal size of crystallites was found to be around 24.46 nm^36^. Calcination is the process of high temperature heating, for the activation of natural clays and stony powdered materials, to enhance the catalytic potentials and adoption properties. The high temperature heating helps to reduce the overall moisture contents and liberate the entrapped associated gases from the deeper layers of material. The structural properties and morphological characteristics of the bentonite clay can significantly be improved by high temperature heating^37^. In the present study, the calcinized bentonite showed more sharp and clear peaks with less background noises as compared to the un-calcinized bentonite. This calcinized bentonite showed characteristic peaks at angle 2θ as follows: 21.03° (110), 26.77° (210), 36.84° (124) and 50.26° (144). These diffraction angles and hkl planes were in close agreement with the standard JCPDS Card No. 01-088-0891. The average crystallite size of calcinized bentonite as calculated by the Debye Sherrer Formula was 25.59 nm^36^. The modification of bentonite clay by the potassium ferricyanide has proved to be helpful in altering the structural properties, crystal lattices and catalytic potentials of composite material. In a recent study,^38^ reported the use of bentonite and potassium ferricyanide as a catalyst, for the cost efficient production of biodiesel. In the XRD spectrum of bentonite and potassium ferricyanide, some sharp and pointed peaks were obtained at the angle 2θ: 21.76°, 26.79°, 27.96°, 29.29°, 34.35°, 55.24° and 60.02° corresponding to the following hkl planes: (110), (220), (210), (124), (400), (144), (600) and (440). These results are in close agreement with the internally available standard compounds under JCPDS Card No. 01-088-0891 and 73-0689. As per the Debye Sherrer Formula, the average crystal size of modified bentonite sample was around 33.76 nm^36,39^. In the present study, the combination of calcinized bentonite and potassium ferricyanide showed no significant variations as compared to the un-calcinized modified bentonite clay. Only the slight differences appeared in the clarity and sharpness of peaks as calcination improves the purity of sample by removing the moisture and entrapped gaseous molecules. Thus, the XRD spectrum of calcinized bentonite and potassium ferricyanide showed peaks at angle 2θ: 16.40°, 24.90°, 26.86°, 30.10°, 31.62°, 35.29°, 39.71° and 54.41° corresponding to the following hkl planes such as (110), (200), (220), (210), (124), (400), (420), (144) and (600). These results are in close agreement with the international standards under JCPDS Card No. 01-088-0891 and 73-0689. The average crystallite size of the samples was found to be around 41.05 nm^36,39^.

**Table 3:** The XRD spectral details of bentonite.

Sr. no	Pos. [°2Th.]	FWHM left [°2Th.]	Area [cts*°2Th.]	Backgr. [cts]	d-spacing [Å]	Height [cts]	Rel. Int. [%]	Particle size (nm)	Planes (hkl)
1	21.4256	0.09	4.16	24.34	4.14392	34.66	33.92	71.88	(110)
2	27.2009	0.1378	13.88	21.66	3.2785	102.17	100	36.98	(210)
3	28.415	0.4723	8.24	21.49	3.14112	17.68	17.3	10.33	–
4	30.0113	0.2362	6.37	21.59	2.97758	27.34	26.76	19.55	(124)
5	45.9004	0.9446	7.37	18.15	1.97711	7.91	7.74	3.19	–
6	60.4426	0.4723	5.68	19.24	1.53164	12.19	11.93	4.85	(144)

Average particle size: 24.46 nm.

[JCPDS File (Card No. 01–088-0891)].

**Table 4:** The XRD spectral details of calcinized bentonite.

Sr. no	Pos. [°2Th.]	FWHM left [°2Th.]	Area [cts*°2Th.]	Backgr. [cts]	d-spacing [Å]	Height [cts]	Rel. Int. [%]	Particle size (nm)	Planes (hkl)
1	12.6364	0.1181	5.7	85.6	7.0053	48.97	19.22	92.87	–
2	21.0349	0.2362	9.02	42.92	4.2235	38.73	15.21	27.89	(110)
3	26.7689	0.0787	19.78	39.42	3.33041	254.75	100	65.79	(210)
4	28.0402	0.2362	9.42	38.08	3.18224	40.42	15.87	20.93	–
5	29.5957	0.1574	8.49	36.83	3.01843	54.69	21.47	29.75	–
6	34.7395	0.9446	11.46	28.92	2.58238	12.29	4.83	4.22	–
7	36.8365	0.4723	7.18	27.85	2.44005	15.42	6.05	7.97	(124)
8	39.653	0.2362	4.81	27.33	2.27299	20.67	8.11	14.79	–
9	45.5482	0.9446	7.82	25.98	1.99158	8.4	3.3	3.22	–
10	50.2641	0.2362	4.98	26.05	1.81522	21.37	8.39	11.67	(144)
11	60.0902	0.9446	9.33	27.72	1.53977	10.01	3.93	2.44	–

Average particle size: 25.59 nm.

[JCPDS File (Card No. 01-088-0891)].

**Table 5:** XRD spectral details of bentonite and potassium ferricyanide.

Sr. no	Pos. [°2Th.]	FWHM left [°2Th.]	Area [cts*°2Th.]	Backgr. [cts]	d-spacing [Å]	Height [cts]	Rel. Int. [%]	Particle size (nm)	Planes (hkl)
1	21.7657	0.1574	7.04	35.57	4.08332	45.34	45.51	40.46	(110)
2	26.7885	0.1378	12.47	31.34	3.32802	91.77	92.11	37.55	(220)/(210)
3	27.9614	0.4723	7.31	31.04	3.19103	15.68	15.74	10.49	–
4	29.292	0.0787	7.74	30.7	3.04903	99.63	100	60.12	–
5	34.3533	0.1181	6.87	27.17	2.61052	58.95	59.17	34.16	(124)/(400)
6	55.2429	0.09	5.1	27.52	1.66146	42.48	42.64	27.88	(144)/(600)
7	60.0228	0.09	4.28	26.37	1.54007	35.63	35.76	25.66	(440)

Average particle size: 33.76 nm.

[JCPDS File (Card No. 01-088-0891)].

[JCPDS File (Card No. 73-0689)].

**Table 6:** XRD spectral details of calcinized bentonite and potassium ferricyanide.

Sr. No	Pos. [°2Th.]	FWHM Left [°2Th.]	Area [cts*°2Th.]	Backgr. [cts]	d-spacing [Å]	Height [cts]	Rel. Int. [%]	Particle Size (nm)	Planes (hkl)
1	16.4068	0.09	4.99	67.39	5.39849	41.61	42.3	93.86	(110)/(200)
2	24.9019	0.2362	7.06	46.69	3.57571	30.29	30.79	23.56	(220)
3	26.857	0.0984	9.55	47.37	3.31968	98.37	100	52.45	(210)
4	30.1015	0.1574	8.45	46.89	2.96886	54.4	55.3	29.25	–
5	31.6221	0.2362	5.81	42.7	2.82948	24.96	25.37	18.56	–
6	35.2871	0.09	5.52	47.04	2.54145	45.96	46.72	43.64	(124)/(400)
7	39.7085	0.09	4.63	39.38	2.26806	38.62	39.26	38.78	(420)
8	54.4065	0.09	7.46	35.85	1.68501	62.15	63.18	28.31	(144)/(600)

Average particle size: 41.05 nm.

[JCPDS File (Card No. 01-088-0891)].

[JCPDS File (Card No. 73-0689)].

## Conclusions

Following important conclusions can be withdrawn from the present study. Plum seed oil is of toxic nature and can be added to list of those waste oils which can be further explored to produce biodiesel. The present study reported the use of Bentonite–potassium ferricyanide, White pocha-potassium ferricyanide, Granite-potassium ferricyanide, Sindh clay-potassium ferricyanide, and Kolten-potassium ferricyanide composites to produce biodiesel from plum seed oil. The maximum biodiesel yield for all composite catalysts was obtained at 0.30% catalyst concentration and 60 °C. The maximum biodiesel yield was recorded for Bentonite–potassium ferricyanide composite which further increased after calcination of the composite. The fuel quality parameters of all biodiesel samples were found in the standard range. The calcination process was remarkably effective in the removal volatile compounds from composite materials to generate further active sites to enhance biodiesel yield. FTIR indicated the presence of surface hydroxyl groups and bonded water on the surface of composite materials. Calcinized bentonite–potassium ferricyanide composite found to contain more nano rod like structures at its surface as compared to Bentonite–potassium ferricyanide composite which contained spherical particles. EDX data of Bentonite–potassium ferricyanide composite and Calcinized bentonite–potassium ferricyanide composite show that after calcination carbon and oxygen was reduced. The other lost volatile compounds after calcination were of Na, Mg, Al, Si, and S.

### Statement

It is submitted that the experimental research on plants, including the collection of plant material, complied with relevant institutional, national, and international guidelines and legislation.
